# Supplementary figures and images for: Plasticity of gene expression in the nervous system by exposure to environmental odorants that inhibit HDACs
Source: eLife. 2024 Feb 27;12:RP86823. doi: 10.7554/eLife.86823 (PMC10942631; doi:10.7554/eLife.86823)

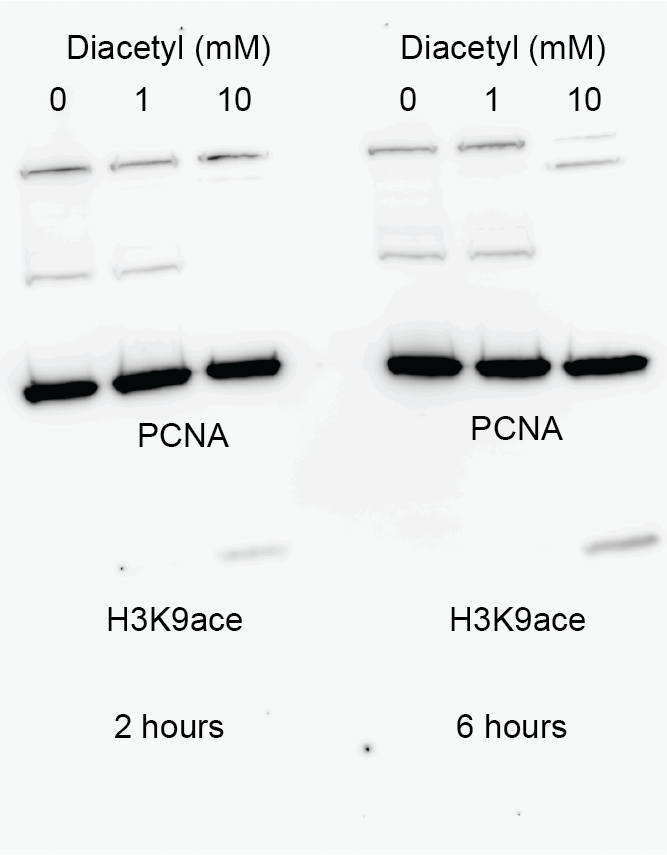

Supplement: Figure 2—source data 1. [file elife-86823-fig2-data1.zip › Fig2AB-H3K9ace-labeled.png]

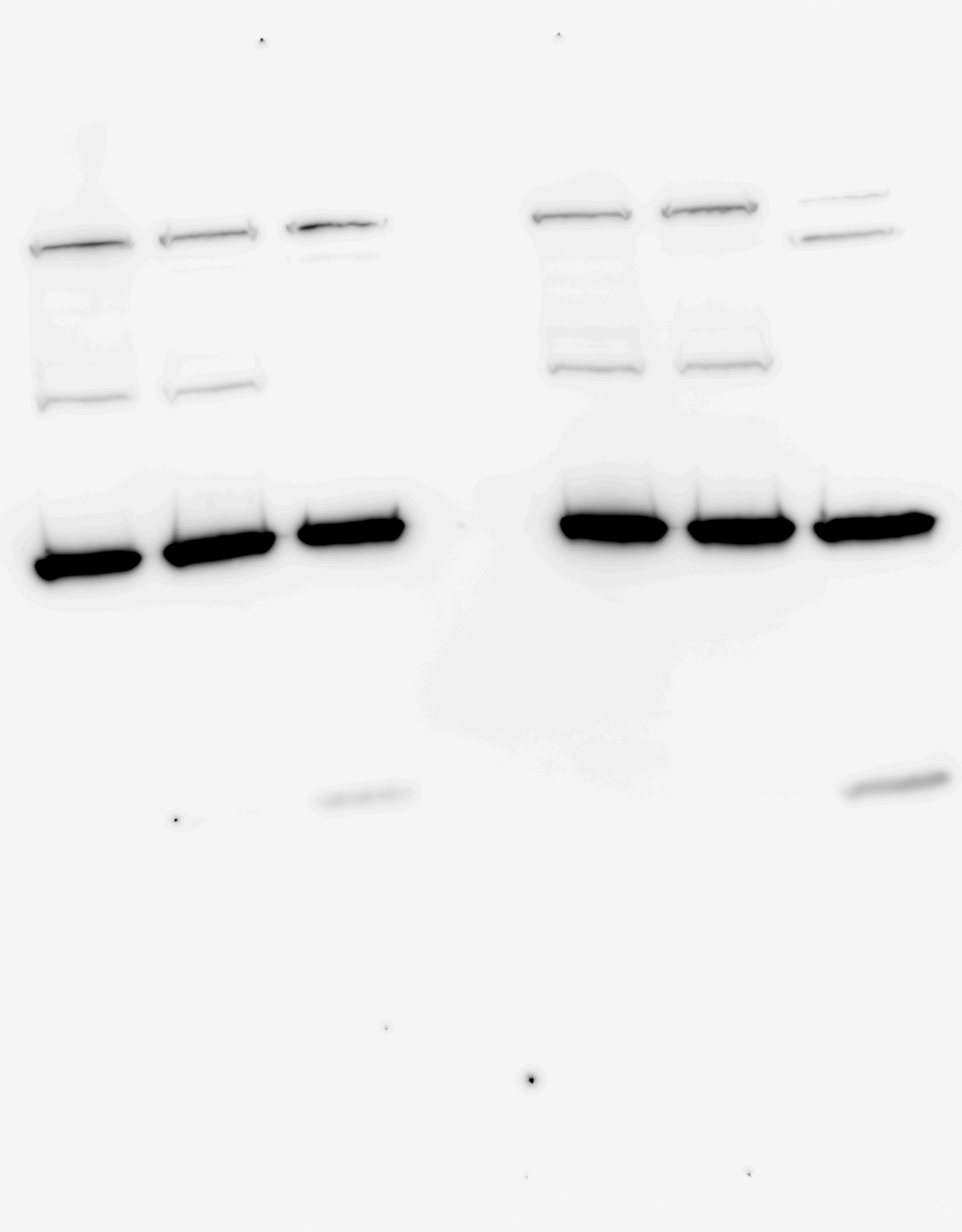

Supplement: Figure 2—source data 1. [file elife-86823-fig2-data1.zip › Fig2AB-H3K9ace-ori.tif]

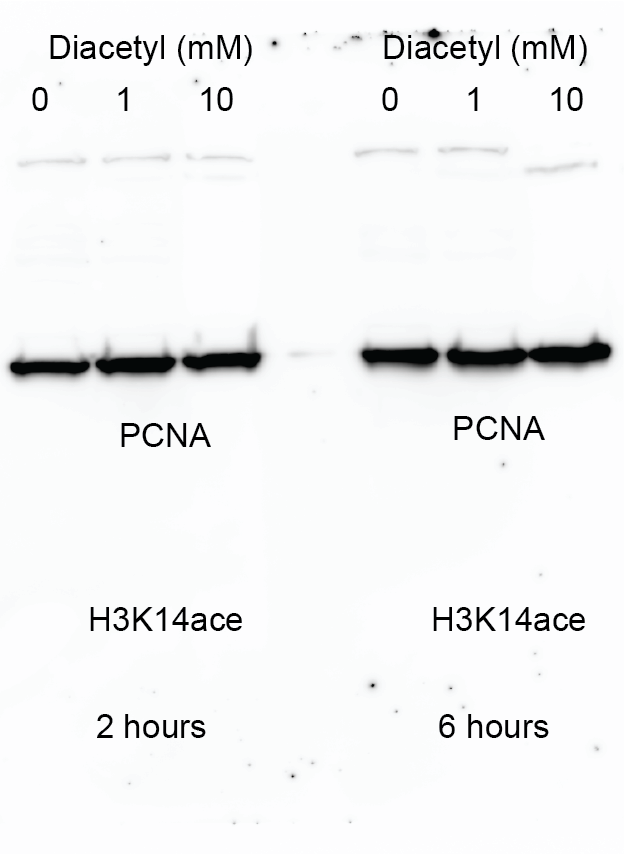

Supplement: Figure 2—source data 2. [file elife-86823-fig2-data2.zip › Fig2AB-H3K14ace-labeled.png]

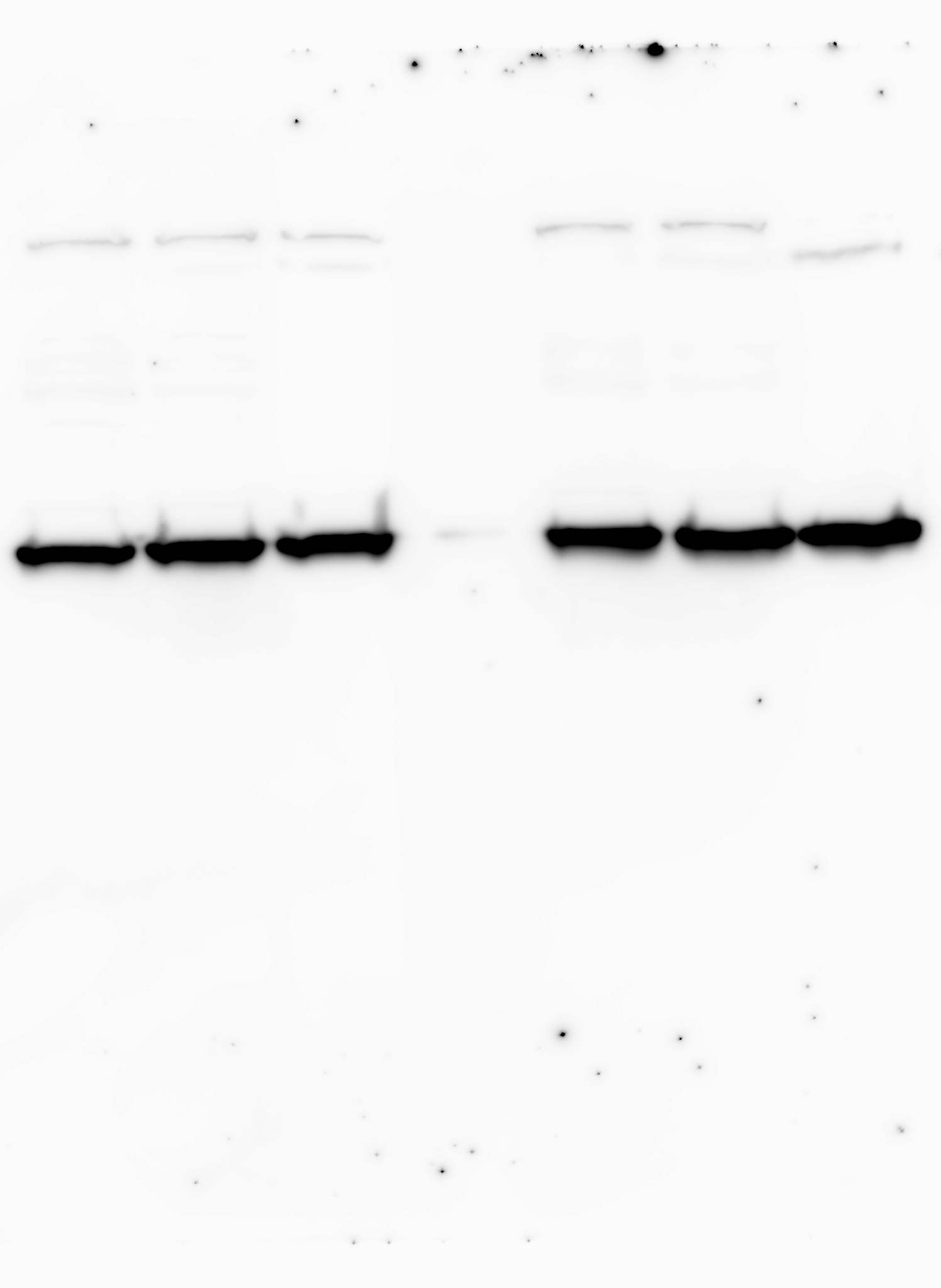

Supplement: Figure 2—source data 2. [file elife-86823-fig2-data2.zip › Fig2AB-H3K14ace-ori.tif]

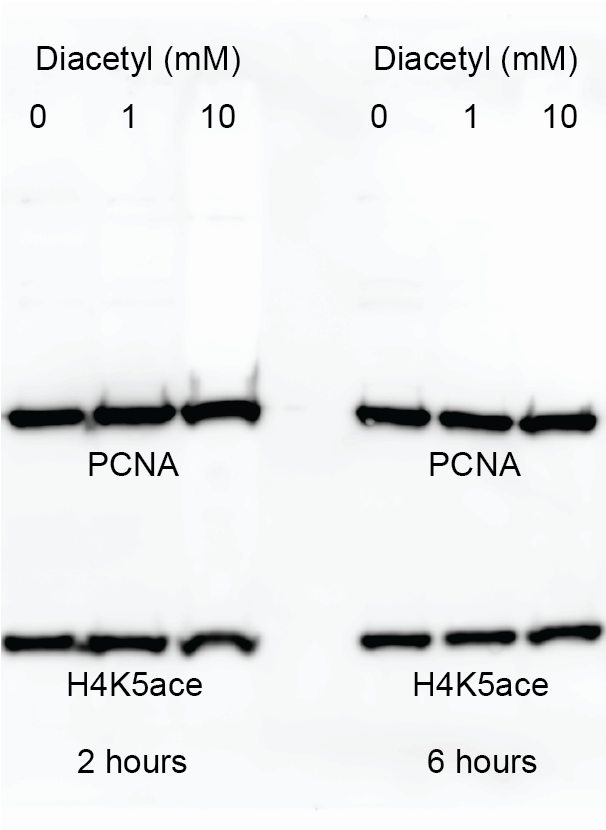

Supplement: Figure 2—source data 3. [file elife-86823-fig2-data3.zip › Fig2AB-H4K5ace-labeled.png]

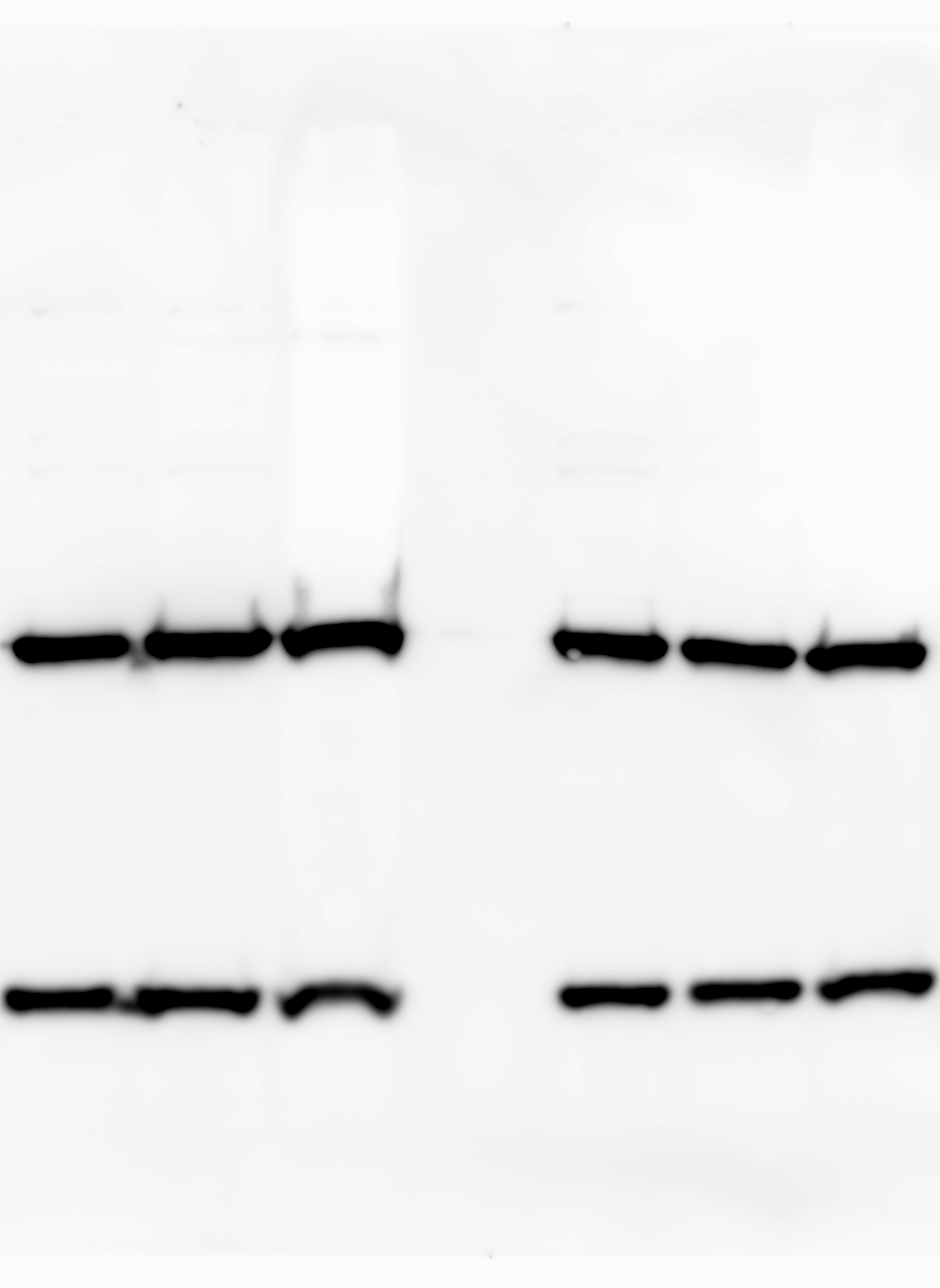

Supplement: Figure 2—source data 3. [file elife-86823-fig2-data3.zip › Fig2AB-H4K5ace-ori.tif]

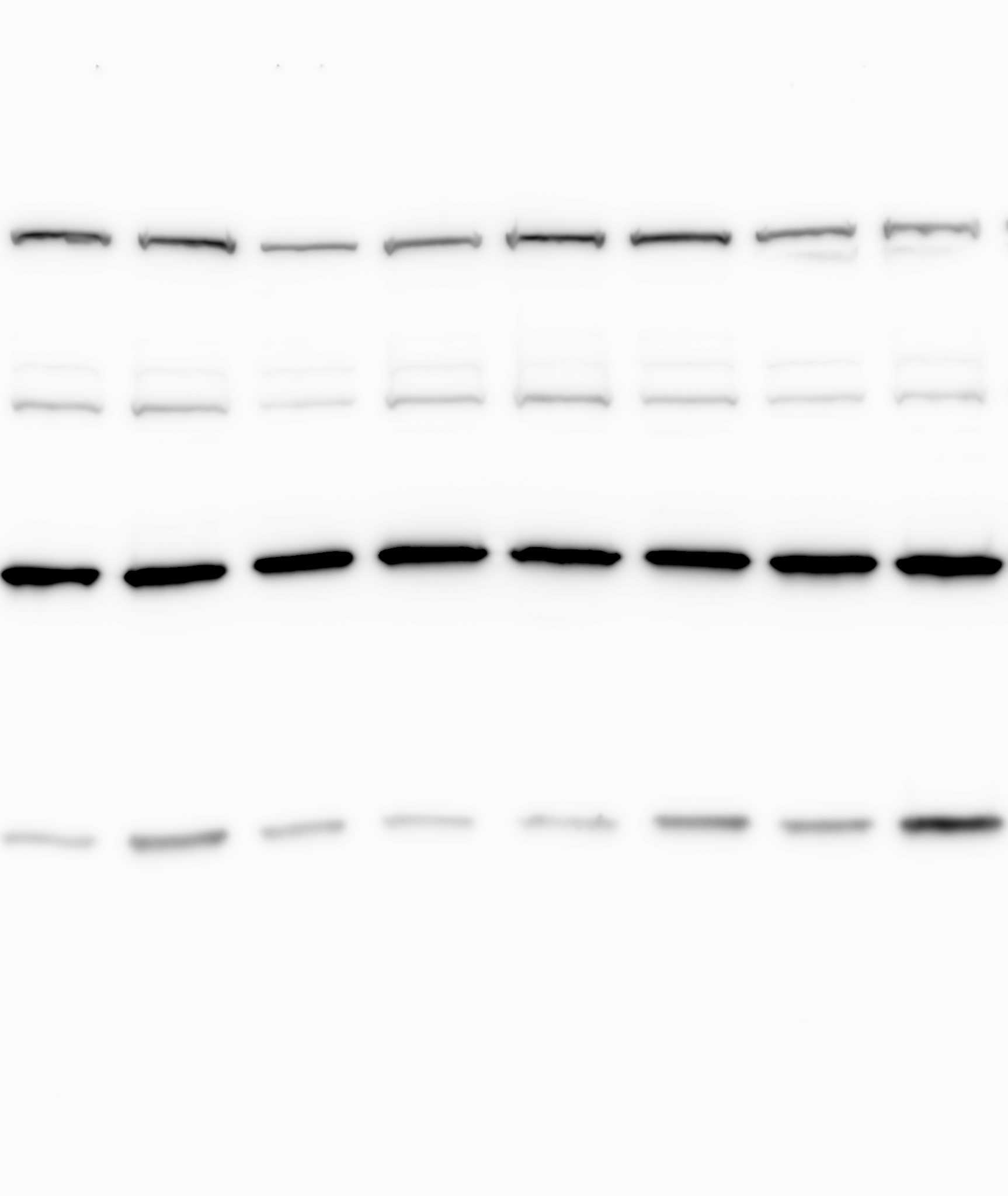

Supplement: Figure 2—source data 4. [file elife-86823-fig2-data4.zip › Fig2C-H3K9ace-ori.tif]

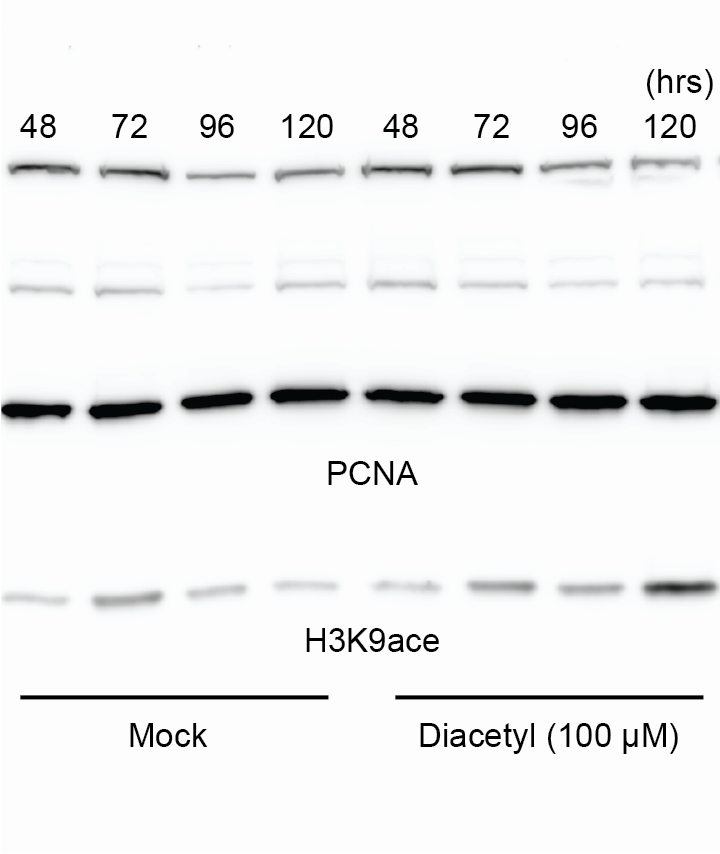

Supplement: Figure 2—source data 4. [file elife-86823-fig2-data4.zip › Fig2C-H3K9ace-labeled.png]
