# Supplementary material for: Plasticity of gene expression in the nervous system by exposure to environmental odorants that inhibit HDACs
Source: eLife. 2024 Feb 27;12:RP86823. doi: 10.7554/eLife.86823 (PMC10942631; doi:10.7554/eLife.86823)
Supplement: Figure 3—source data 1. [file elife-86823-fig3-data1.docx]

| **#** | **Common sources of Diacetyl** | **AVG Diacetyl (ppm)** | **References** |
| --- | --- | --- | --- |
| **1** | Starter Distillates (SDL) in Dairy Product Production | 1.2-22,000 | Rincon-Delgadillo, M. I., Lopez-Hernandez, A., Wijaya, I., & Rankin, S. A. (2012). Diacetyl levels and volatile profiles of commercial starter distillates and selected dairy foods. *Journal of dairy science*, *95*(3), 1128-1139. |
| **2** | Mainstream cigarette smoke | 250-361 | Pierce, J.S., et al., *Diacetyl and 2,3-pentanedione exposures associated with cigarette smoking: implications for risk assessment of food and flavoring workers.* Crit Rev Toxicol, 2014. **44**(5): p. 420-35. |
| **3** | Microwave Popcorn Facility | 1-57.2 | Kanwal, R., et al., *Occupational Lung Disease Risk and Exposure to Butter-Flavoring Chemicals After Implementation of Controls at a Microwave Popcorn Plant.* Public Health Reports, 2011. **126**(4): p. 480-494. |
| **4** | Baked goods | 44 | Hall, R.L. and B.L. Oser, *Recent Progress in Consideration of Flavoring Ingredients under Food Additives Amendment .3. Gras Substances.* Food Technology, 1965. **19**(2p2): p. 151-&. |
| **5** | Candy | 21-35 | Hall, R.L. and B.L. Oser, *Recent Progress in Consideration of Flavoring Ingredients under Food Additives Amendment .3. Gras Substances.* Food Technology, 1965. **19**(2p2): p. 151-&. |
| **6** | Gelatins/Puddings | 19 | Hall, R.L. and B.L. Oser, *Recent Progress in Consideration of Flavoring Ingredients under Food Additives Amendment .3. Gras Substances.* Food Technology, 1965. **19**(2p2): p. 151-&. |
| **7** | Unflavored Brewed cup of Coffee | 7 | Pierce, J.S., et al., *Characterization of naturally occurring airborne diacetyl concentrations associated with the preparation and consumption of unflavored coffee.* Toxicol Rep, 2015. **2**: p. 1200-1208.  Yeretzian, C., A. Jordan, and W. Lindinger, *Analysing the headspace of coffee by proton-transfer-reaction mass-spectrometry.* International Journal of Mass Spectrometry, 2003. **223**(1-3): p. 115-139. |
